# Effect of chronic exercise on fluoride metabolism in fluorosis-susceptible mice exposed to high fluoride

**DOI:** 10.1038/s41598-018-21616-2

**Published:** 2018-02-16

**Authors:** Sandra L. Amaral, Liane B. Azevedo, Marilia A. R. Buzalaf, Mayara F. Fabricio, Mileni S. Fernandes, Ruth A. Valentine, Anne Maguire, Fatemeh V. Zohoori

**Affiliations:** 1 Department of Physical Education, Science Faculty, São Paulo State University, Bauru, São Paulo 17033-360 Brazil; 2 School of Health and Social Care, Teesside University, Middlesbrough, TS1 3BA UK; 3 Bauru School of Dentistry, University of São Paulo, Bauru, SP 17012-901 Brazil; 4 Centre for Oral Health Research, School of Dental Sciences, Newcastle University, NE2 4BW, Newcastle-upon-Tyne, NE2 4BW UK

## Abstract

The present study investigated the effect of chronic exercise on fluoride (F) metabolism in fluorosis-susceptible mice exposed to high-F and explored the relationship between F concentrations in bone and plasma. Thirty male mice were randomised into three groups: Group I (No-F, No-Exercise), Group II (50 ppmF, No-Exercise), Group III (50 ppmF, Exercise). Body weight and physical performance of all mice were measured at baseline and end of experiment. F concentrations of plasma and bone were measured at the end of experiment. Mean plasma F concentration was significantly higher (p < 0.001) in Groups II and III compared with Group I. Mean bone F concentration was also significantly higher (p < 0.01) in Groups II and III compared with Group I. There was a significant correlation (p = 0.01, r = 0.54) between F concentration of plasma and bone. Mean body weight of Group I mice was significantly higher than Group II (p < 0.001) and Group III (p = 0.001) mice at the end of the experiment. This study, which provides the first data on the effect of chronic exercise on F metabolism in fluorosis-susceptible mice, suggests no effect of chronic exercise on F in plasma and bone. However, exposure to high-F resulted in lower body weight and exercise capacity in mice.

## Introduction

Fluoride (F) is considered a beneficial nutrient, present in trace amounts in the body^1^ and of public health significance due to its role in mineralisation of both teeth and bones. The effectiveness of topical exposure to even low F concentrations (<0.02 ppm) in providing benefit in the prevention of dental caries has been well defined along with the risk of dental and skeletal fluorosis as side effects of excessive exposure to systemic F during the developmental phases of teeth and bone. However, the bioavailability of F, and consequently its body retention, is more important than F intake in the development of dental and skeletal fluorosis. To avoid or minimise F side effects, it is important to characterise the physiological effects of F through improved understanding of its metabolism. Several factors, including level of F exposure, stage of skeletal development, acid-base balance, genetics and exercise have been suggested to affect F metabolism and body retention^2^.

Among the factors influencing F metabolism, little evidence is available on the effects of exercise and genetics on F metabolism and body retention. Due to the well-recognised multiple health benefits of exercise, many community- and school-based interventions have been implemented globally to increase the level of physical activity in populations^3^. At the same time, a number of F-based community prevention programmes, such as community-based fluoridated salt and school-based fluoridated milk schemes are currently operating across the world, to help improve oral health by reducing the risk of dental caries, particularly in children^4^. Changes in physiological responses to exercise may impact the pharmacokinetics of F, which could be important in terms of the impact of F on tooth and bone development and the timing of F ingestion when used as a dental caries preventive therapy.

Ingested F is rapidly absorbed from the stomach and proximal small intestine and it circulates in the body via plasma which acts as a central compartment for F distribution to hard and soft tissues and for urinary F excretion, which is the main route for elimination of F from the body. At least 99% of F in the body is associated with calcified tissues^5^ and F is one of only a few known agents that can stimulate osteoblast proliferation^1^; it demonstrates a biphasic dose relationship, being mitogenic to osteoblastic precursors at low doses and inhibitory to osteoclasts at high doses^6^. Exercise has been linked with a reduction in expression of osteocytes but an increase in expression of osteoblasts^7^.

A recent human experimental study reported higher plasma F concentrations with moderate and vigorous intensity exercise compared with a non-exercised control in young adults after an acute session of exercise at different intensities, as well as reduction in urinary F excretion after moderate intensity exercise compared with the control group^8^. However, two studies with rats exposed to a treadmill-based exercise regime with running speed set as 18 m/min and 2.25 m/min respectively for 1 h, reported a significant reduction in plasma F concentration after this exercise^5,9^. A significantly higher bone F content in rats exercised for 30 days has also been reported^9^.

A considerable variation in dental and skeletal fluorosis prevalence and severity is reported among and within populations^10^ which cannot be fully accounted for by F “dose” suggesting that other factors contribute to the body’s response to F exposure. Resistance or susceptibility to fluorosis appears to be additionally influenced by host (e.g. genetics) and environmental (e.g. exercise) factors and their interactions^9,11^. Therefore, studies looking at the effect of environmental factors on F metabolism should also take into consideration the possible effect of genetics; i.e. gene-environment interactions.

Among commonly-used laboratory animals, inbred mice have been the species of choice to study the effect of environmental factors on F metabolism because of their strain-dependent responses to F in the development of dental fluorosis. Inbred mouse strains are usually categorised into three groups according to their resistance/susceptibility to F in terms of development of dental fluorosis: F-resistant (e.g. 129P3/J), intermediate (e.g. SWR/J) and F-susceptible (e.g. A/J) strains^12^. By selecting a specific strain (i.e. either 129P3/J or A/J), any effects of genetic differences would be moderated.

Very little is known about the effect of exercise on F metabolism and body F retention and there are no data on the effect of exercise on F retention in fluorosis-susceptible mice (A/J). Therefore the main aim of this study was to investigate the effects of chronic exercise at high intensity on F metabolism in fluorosis-susceptible mice (A/J) exposed to high F through the water in their diet. The objectives were to measure and compare exercise capacity,  plasma and bone (femur) F concentrations in mice with- and without exercise. The subsidiary aim was to explore the relationship between femur and plasma F in these mice.

## Material and Methods

### Study design

The experiment was carried out using fluorosis-susceptible (A/J) mice. All procedures were approved by the Ethics Committee for Animal Experiments of Bauru Dental School, USP (#009-2015) and all experiments were performed in accordance with relevant guidelines and regulations. Thirty male weanling mice (21 days old) were obtained from vivarium of Bauru School of Dentistry. All mice were kept in pairs in plastic cages in a climate-controlled room, at Bauru Dental School, University of Sao Paulo, with a 12 h light/dark cycle maintained at 23 ± 1 °C and 40–80% humidity. All three groups of animals had *ad libitum* access to low-F food (Presence, Purina, <1 mg/kg F).

The animals were randomly distributed into three groups, according to the F concentration of water received and their exercise regime: Group I (Control): No Fluoride (0 ppmF) and No Exercise; Group II: Chronic F exposure (50 ppmF) and No Exercise, and; Group III: Chronic F exposure (50 ppmF) and Exercise. After one week of adaptation to the environment (week 0- no F ingestion and no exercise), experimental Groups II and III received drinking water containing 50 ppm F ion (as sodium fluoride), while Group I received deionised water, all for 11 weeks. After 3 weeks of the F feeding routine, animals in Group III began their exercise training regime, which consisted of High-Intensity Interval Training (HIIT) on a treadmill 5 days per week, for 8 weeks (i.e. week 4 to week 11 inclusive). Throughout the 11-week period (3 weeks feeding routine and 8 weeks training), the body weight of all animals was measured weekly and water consumption per cage was recorded daily.

The staff who administered the drinking-water and diet of the mice along with their exercise regime differed from those staff assessing the effects. Mice and their subsequent plasma and bone samples were labelled such that staff analysing the samples and the results were unaware of the type of treatment (i.e. diet and exercise) received.

### Exercise protocol

The exercise protocol was piloted with four male mice to inform the methodology for the current study. The pilot study revealed that the HIIT protocol for mice previously suggested in the literature^13^ was not feasible for the present study and the mice were unable to complete the distance of 1000 m, following interval sessions of 2 minutes at 90% and 1 minute at 40% of maximum running speed. Therefore, the intensity and duration of the exercise intervals were adjusted to 1 minute at 80% of maximal running speed, followed by a 3-minute recovery (rest) period which enable the mice to cover the distance of 1000 m as suggested in the original protocol^13^.

#### Determination of maximum running speed

To determine the maximum running speed of the mice, all 30 mice were familiarized with the treadmill for 10 min/session at a speed of 8 m/min and 0% gradient once a day for five days^14^. After the familiarization period, each mouse performed a maximum running test on a treadmill^15^. The test began at 6 m/min with 0% gradient and increased by 3 m/min every 3 minutes thereafter until exhaustion (i.e. when the animal could no longer run). This maximum running test was repeated for each mouse in all 3 groups after 4 weeks, to allow for any adjustment of exercise intensity (if needed) and again at the end of the experimental period to measure the effect of training on maximum running speed.

#### Exercise training protocol

The Group III mice were submitted to a High-Intensity Interval Training routine, 5 days per week over the 8 week experimental period (i.e. week 4 to week 11 inclusive). The HIIT sessions consisted of a 5 min warm-up at 40% of each mouse’s maximum running speed followed by a sequence of nineteen short periods (1 minute each) of high intensity effort, at 80% of maximal running speed, followed by a 3 minute recovery period (rest). Each training session ended when the mouse completed a distance of 1000 meters. The training sessions were performed at the exact same time each weekday to avoid diurnal effects on training performance^16^.

### Sample preparation and fluoride analysis

After 11 weeks, animals were anaesthetised with ketamine/xylazine and a blood sample collected from the heart into a lightly heparinised syringe. The animals were then killed by excessive anaesthesia and their right femurs were removed.

Blood samples were centrifuged at 1400 × g; plasma was separated and stored at −20 °C. The bone (femur) samples were ashed at 600 °C overnight in a muffle furnace (Fornitec, model HW1000; Fornitec Industria e Comercio, Santo Amaro, Sao Paulo, Brazil), and the ash weighed and stored at room temperature prior to F analysis^9,17^.

Plasma (ng/ml) and femur (µg/g ash) F concentrations were determined in duplicate after overnight hexamethyldisiloxane (HMDS)-facilitated diffusion^18,19^ using a F-ion-specific electrode (Orion Research, Model 9409) and a miniature calomel electrode (Accumet, #13-620-79) both coupled to a potentiometer (Orion Research, Model EA 940). Fluoride standards (0.005 to 0.19 µg F for plasma and 1.9 to 38.0 µg F for bone) were prepared in triplicate and diffused in the same manner as the samples. In addition, non-diffused standards were prepared with exactly the same F concentrations as the diffused standards. Comparison of the mV readings demonstrated that the F in the diffused standards had been completely trapped and analysed (recovery > 95%). The mV potentials were converted to µg F using a standard curve with a correlation coefficient of r ≥ 0.99.

### Statistical analysis

#### Sample size

A previous study by Lombarte and co-workers with a sample size of 6 rats per group, submitted to an exercise regime, found that treatment with 15 ppm F significantly increased insulin resistance, which was ameliorated by physical exercise^9^. Likewise, significant differences were obtained in plasma and bone (femur) F concentrations of mice treated with 0 or 50 ppm F in the drinking water when a sample size of 6 was used^11^. However, power analysis based on plasma F concentration of A/J mice, in the study by Carvalho and co-workers^11^, suggested that a sample size of 9 mice per treatment was needed to obtain 80% power, at a significance level of 0.05, for plasma F concentrations (effect sizes 6.9 ng/ml and 97.9 ng/ml respectively for comparison of control (0 ppm) with high (50 ppm) F treatments). In view of this, 10 mice per group were used in the present study.

#### Data analysis

Data were analysed using Sigma Stat version 3.11 software. All data were normally distributed and all values are presented as means and standard error of the means (SEM). Statistically significant differences among all groups were detected using ANOVA. Statistically significant differences were further investigated using a post-hoc test (Tukey) with statistical significance set at α < 0.05. Pearson correlation was used to investigate the relationship between F concentrations of plasma and bone.

### Data Availability

The datasets generated during the current study are available from the corresponding author on reasonable request.

## Results

Two mice from Group II (50 ppmF, No-exercise) and 4 mice in Group III (50 ppmF, Exercise) did not complete the running distance (i.e. 1000 m) during the training sessions and therefore were excluded from the experiment. Results of the maximum running speed test for the 24 mice that completed the experiment, are presented in Table 1, according to exercise regime. There was no significant difference in the maximum running speed between groups at baseline. However, at the end of experiment, the maximum running speed was significantly (p = 0.012) increased in Group III mice (50 ppmF; Exercise) which had undergone HIIT, compared with baseline (+5.5 m/min), whereas the corresponding value was statistically significantly (p = 0.005) decreased in Group I (−4.5 m/min) and Group II (−10.9 m/min) mice which had not undergone HIIT. As presented in Table 1, there was also a statistically significant difference in the ‘change in running speed’ between the groups: Group I vs. Group II (p = 0.048), Group I vs. Group III (p = 0.004), and Group II vs. Group III (p < 0.001).

**Table 1:** Mean (SEM) maximal running speed (m/min) at baseline, end of experiment and the change in running speed between baseline and end of experiment.

Group	No	Mean (SEM) maximum running speed (m/min)		Change (speed at the end of experiment − speed at baseline) (m/min)	
		Baseline (week 3)	End of experiment (week 11)	Mean (SEM)	P value
I (Control)	10	24.0 (1.2)	19.5 (0.9)	−4.5 (1.2)	0.005
II (50 ppmF, no-exercise)	8	22.9 (1.6)	12.0 (1.7)	−10.9 (2.6)^a^	0.005
III (50 ppmF, exercise)	6	21.5 (1.8)	27.0 (2.0)	+ 5.5 (1.4)^b,c^	0.012

P-values using one-way ANOVA followed by a Tukey’s post-hoc analysis test:

^a^Group I vs Group II: p = 0.048.

^b^Group I vs Group III: p = 0.004.

^c^Group II vs Group III: p < 0.001.

There was no statistically significant difference in mean (SEM) daily water consumption per mouse across the whole of the experimental period: 3.92 (0.14) ml for Group I (Control), 3.84 (0.11) ml for Group II (50 ppmF, No-exercise) and 3.86 (0.11) ml for Group III (50 ppmF, Exercise).

**Figure 1:**
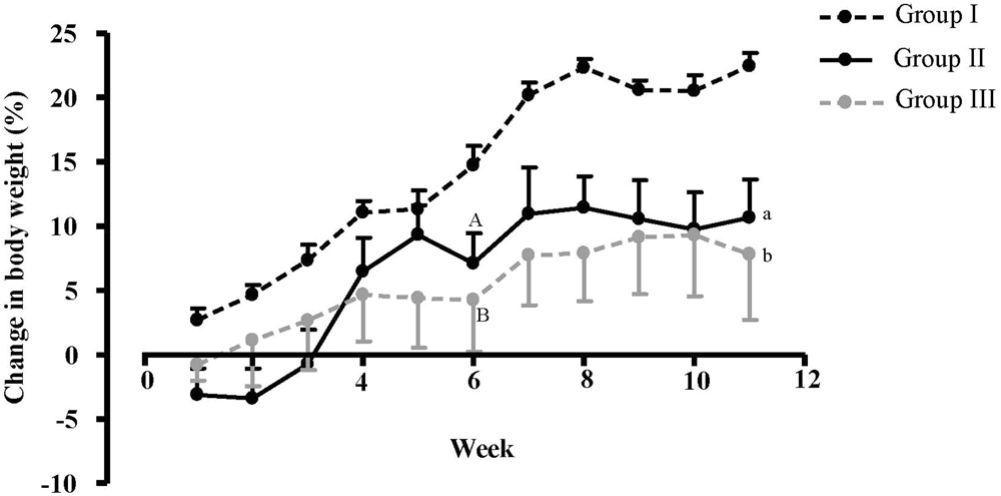
Mean changes in body weight (g), compared with baseline weight (week 0), for mice in Group I (Control, n = 10), Group II (50 ppm F, No-exercise; n = 8) and Group III (50 ppm F, Exercise; n = 6). ^A^Group II vs Group I at 6 weeks: p = 0.013. ^B^Group III vs Group I at 6 weeks: p = 0.039. ^a^Group II vs Group I at 11 weeks: p < 0.001. ^b^Group III vs Group I at 11 weeks: p = 0.001.

Mean (SEM) body weight of mice at baseline, before starting the experiment, was similar among the groups: 22.83 (0.39) g, 21.63 (0.67) g and 22.37 (0.50) g for Group I (Control), Group II (50 ppmF, No-exercise) and Group III (50 ppmF, Exercise), respectively. The corresponding figures at the end of experiment were 29.30 (0.47) g, 24.54 (1.20) g and 24.65 (1.39) g, respectively, with the percentage changes in body weight throughout the 11 weeks of the experiment differing among the three groups. As Fig. 1 shows the increase (%) in body weight of Groups II and III mice was significantly less than Group I (Control) mice by week 6 (Group II vs Group I p = 0.013 and Group III vs Group I p = 0.039). Thereafter the increase in body weight of Group I mice (Control) remained significantly greater than the other two groups until the end of experiment (Group II vs Group I p < 0.001; Group III vs Group I p = 0.001), while there was no difference in change in body weight between the two groups exposed to 50 ppm F (Group II and III) throughout the experiment.

**Figure 2:**
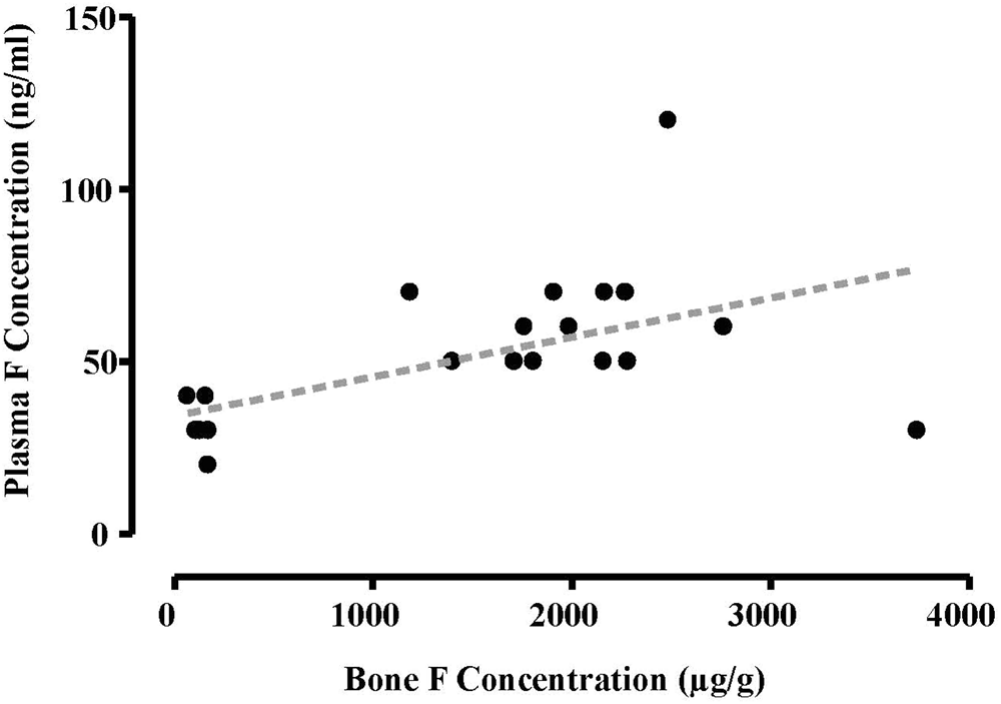
Relationship between plasma F concentration (ng/ml) and bone F concentration (µg/g femur ash) for mice in the three groups combined (r = 0.54, p = 0.01). “Plasma F concentration (ng/ml) = 33.43 + (0.012 × Bone F concentration (µg/g)”.

Table 2 presents the mean (SEM) F concentrations in plasma (ng/ml) and bone (µg/g) for all three groups and shows significantly higher concentrations in the groups exposed to 50 ppm F (Groups II and III) compared with Group I, and especially for bone F (p < 0.001), but no differences between Groups II and III in terms of the impact of chronic exercise on body F. There was a significant moderate correlation (p = 0.01, r = 0.54) between F concentration of plasma and bone (Fig. 2): Plasma F concentration (ng/ml) = 33.43 + (0.012 × Bone F concentration (µg/g)).

**Table 2:** Mean (SEM) F concentrations of plasma (ng/ml) and bone (µg/g) in Group I (Control), Group II (50 ppm F, No-exercise) and Group III (50 ppm F, Exercise) at the end of experiment. P values refer to comparisons within columns (One-way ANOVA followed by a Tukey’s post-hoc analysis test).

Group	Plasma F Concentration (ng/ml)	Bone F Concentration (µg/g femur ash)
I (Control)	29.3 (2.57)	137.43 (13.54)
II (50 ppmF, no-exercise)	62.87 (8.55)	2117.62 (155.22)
III (50 ppmF, exercise)	54.76 (6.20)	2116.17 (352.36)
P value	Group I vs Group II: p = 0.002	Group I vs Group II: p < 0.001
	Group I vs Group III: p = 0.037	Group I vs Group III: p < 0.001
	Group II vs Group III: NS	Group II vs Group III: NS

## Discussion

This study provides the first data on the effect of chronic exercise on F metabolism in fluorosis-susceptible mice (A/J) exposed to high F. The findings imply that chronic exercise has no effect on F in plasma and bone of fluorosis-susceptible mice, which may be important in terms of development of appropriate nutrition and exercise strategies for optimising general and oral health in children. However, exposure to high levels of F resulted in a significantly lower body weight as well as lower running performance in mice, although the reasons for this are unclear.

The study was undertaken with one specific strain of mice (A/J - fluorosis-susceptible) to avoid any possible genetic-related variability in F metabolism. With regard to animal welfare and ethics, the number of animals to be used should be minimal with appropriate justification when undertaking any animal study. Therefore, an active choice was made to exclude the use of a ‘No F, plus exercise group’ in order to limit the impact on animal usage and since the planned design in the current study was to compare the effect of F exposure with and without exercise.

The results for exercise performance (Table 1) showed an increase in the maximal running speed on a treadmill in the HIIT trained mice (Group III) receiving chronic exercise compared with the non-trained mice (Group I and Group II), which supports the validity of the exercise protocol for mice, developed for this study. The lack of a statistically significant difference in mean daily water consumption per mouse observed between groups confirms that the burden of F exposure was comparable for Groups II and III.

An interesting observation in this study was that the mean daily water consumption did not increase with the undertaking of an exercise regime in the mice, which contrasts with the increased water consumption with exercise to replace fluid lost in sweat during exercise, seen in humans^20^. This lack of change in water consumption with exercise in mice could be explained by the smaller number of sweat glands found in the mouse, compared with humans, and therefore potentially less water loss during exercise, as well as the differences which exist between mice and humans in terms of their thermo-regulatory mechanisms^21^. In addition, it should be mentioned that A/J mice typically ingest higher amounts of water than other strains of mice, such as 129P3/J mice, regardless of F exposure^11^.

The results of the present study clearly showed higher concentrations of F in plasma and bone of mice exposed to 50 ppmF in water (Groups II and III) compared with those in Group I (Control) with no F intake, but no effect of exercise on the degree of either F absorption or F retention in bone as evidenced by the lack of difference in concentrations of F found in plasma and bone (Table 2) between Group II (50 ppmF, No-exercise) and Group III (50 ppmF, Exercise).

The main limitation of the present study was the number of mice completing the experiment in the exercised group (Group III; n = 6) compared with the comparable non-exercised group (Group II, n = 8) and in view of these findings, further research with additional numbers of mice would be useful. There is no other study in the literature on the effect of chronic exercise on levels of F in plasma and bone in mice for comparison with the present study, however, two rat studies^5,9^ and one human study^8^, report conflicting evidence for the effect of exercise on F metabolism.

The human experimental study^8^ with nine young adults, who took a 1-mg F-tablet before undertaking acute exercise at different intensities, reported higher post-exercise plasma F concentrations with moderate (15.6 ng/ml) and vigorous (14.9 ng/ml) intensity exercise compared with a non-exercised control group (9.6 ng/ml); findings similar to those of the present study: i.e. a slightly, but non-statistically significantly higher, plasma F level in the no-exercise (Group II; 62.87 ng/ml) compared with exercised (Group III; 46.9 ng/ml) mice exposed to 50 ppm F in drinking water. In contrast, two studies with rats^5,9^ reported 58–76% lower plasma F concentrations in exercised rats compared with non-exercised rats, which may be due to differences in exercise regime and F dose used between the two studies. In the earlier study^5^ the rats received a F dose of 5.0 mg/kg by gastric intubation and were exercised on a rotating-drum, with a rotation velocity of 18 m/min, for one hour; whereas in the more recent study^9^, the rats were exposed to a one-hour daily exercise regime at a running speed of 2.25 m/min, on a treadmill, and drank 15 ppm F water *ad libitum* for 30 days. Lombarte and co-workers^9^ found no statistically significant difference in plasma F concentration of control rats exposed to 0 ppmF drinking water compared with non-exercised rats exposed to 15 ppmF water. In addition, they^9^ also reported a significantly higher bone F concentrations in rats exercised for 30 days, in contrast to the present study which showed no significant difference in bone F concentration of mice exercised for 48 days. These conflicting observations on the effect of exercise on plasma F concentration between these studies could be explained by the differences in F exposure (dose and duration of exposure), exercise (intensity and duration of exercise –chronic v acute) and species (rat v mouse v human). Therefore, more studies are needed to look at the effect of exercise on F metabolism using animal models. A mouse-model would be preferable to a rat-model due to the clearly evident differences seen in susceptibility to development of dental fluorosis as well as differences in maximal treadmill running speed and exercise performance according to mouse strain. A weekly increase in running speed of 2.4 m/min during a training period with a running speed of 33 m/min at the end of 8 weeks training has been reported for mice, compared with corresponding figures of 1.8 m/min and 24 m/min in rats^22^.

The present study showed a significant moderate positive correlation (Fig. 2) between F concentrations of bone and plasma indicating that bone F concentration could be predicted from plasma F concentration. This finding is in agreement with the concept of the steady-state relationship existing between concentrations of F in the extracellular fluids (plasma) and the hydration shells of bone crystallites^5^. However, due to the observed moderate correlation and small sample size in the present study, further studies are required to confirm the utility of plasma F as a biomarker for contemporary exposure to F and bone surface F.

Although the present study found no effect of exercise on plasma and bone F, the findings on the effect of F on exercise capacity and body weight of mice very evident. The results of the present study revealed a significant reduction in the mean maximal running speed (m/min) on a treadmill in non-exercised mice exposed to 50 ppm F (Group II), although this negative effect of F was diminished with exercise (Table 1). In addition, although the base line weights were similar between groups, the final mean body weight of the two groups of A/J mice exposed to 50 ppmF, regardless of exercise, was significantly lower (at 24.54 g and 24.65 g for Group II and Group III, respectively) than the control group with no exposure to F (29.30 g). This finding is in agreement with a rat study^23^, which reported a significantly (p < 0.05) lower body weight of rats exposed to 50 ppm F drinking water compared with a control group. It has been suggested that F might induce apoptosis by elevating the expression of B-cell lymphoma 2 (Bcl-2) and Bcl-2-associated protein X (Bax) genes^23,24^.

The lower body weight gain and reduced exercise capacity in the A/J mice exposed to high F might be due to alteration in the expression of liver proteins associated with metabolic processes in response to excessive systemic F. Forty-nine proteins associated with metabolism have been identified with altered expression in male Wistar rats exposed to 50 ppm F, of which nine relate to carbohydrate metabolism and another nine to lipid metabolism^25^. This latter study also reported an overexpression in proteins involved in the beta oxidation of fatty acids, - a multi-step process by which fatty acids are broken down to produce energy, - in the rats treated with 50 ppm F. A study in 10–12 year old children receiving >2 ppmF drinking water in China^26^ also reported a rise in the activity of their serum lactate dehydrogenase (LDH). LDH is an enzyme involved in the simultaneous inter-conversion of pyruvate, a final product of glycolysis, to lactate. Breakdown of tissues, due to exercise, releases LDH in the blood, raising serum LDH^27^, therefore, the lower body weight of mice exposed to high F, in the present study, could be due to the higher rate of tissue-breakdown in the groups exposed to F compared with the control group. The positive effect of HIIT on physical performance of the mice exposed to high F might also be explained by an alteration in their LDH level. During exercise, when oxygen is in short-supply, LDH increases the rate of lactate production. Although lactate is correlated with muscle fatigue, lactate production during exercise is fuel for the brain, heart and skeletal muscle^28^. Lactate production via the LDH complex acts to prevent muscular failure and delays the onset of muscle fatigue^29^. Cytosolic oxidized nicotinamide adenine dinucleotide (NAD+) is generated as a result of the lactate-forming reaction, feeding into the glyceraldehyde 3-phosphate dehydrogenase reaction to support cytosolic redox potential and this consequently provides more energy to muscles by encouraging ATP production. During exercise, the density of the lactate- and H^+^-transporting proteins increase and blood flow is improved resulting in an increased systemic lactate and H^+^ clearance^29^.

The main aim of the present study was to investigate the effect of exercise on F metabolism but not the effect of F on body weight or on exercise capacity, therefore the interpretation of these latter results should be cautious. The limitations of the present study include: i) no quantification of body mass (i.e. measurement of muscle mass, fat mass and bone mass) or measurement of lactate and LDH levels to study how F might affect body weight and exercise capacity; ii) lack of a positive control (i.e. an exercise group with no exposure to F) to investigate the magnitude of any effect of F on physical performance; and iii) no evaluation of bone properties.

Disturbed amelogenesis has been reported to occur at F doses which cause plasma F peaks of approximately 10 µmol/l^30^. The administered F dose, in the present study, was 50 ppm, which is almost equivalent to a human F exposure level of 10 ppm^31^, a dose which elevates plasma F concentrations to 10 µmol/l (i.e. similar to those shown to cause dental fluorosis). However, mild enamel fluorosis in the rat incisor has also been reported with a plasma F concentration as low as 1.5 µmol/l^32^. Further studies are therefore recommended to investigate the effect of different doses of F on body weight and exercise performance.

In conclusion, chronic exercise training (HIIT) was shown to have no effect on F concentration in plasma and bone (and therefore F absorption and retention) in fluorosis-susceptible mice (A/J) chronically exposed to a high dietary F dose. The observed lower body weights of mice exposed to high F compared with the control group and the reduced physical performance of non-exercised mice exposed to high F need to be investigated further.
